# Feasibility Study on the Bamboo Grid Instead of Geogrid for Soil–Rock Mixture Subgrade Reinforcing

**DOI:** 10.3390/ma15124047

**Published:** 2022-06-07

**Authors:** Yong Hu, Shanling Chen, Cekun Xie, Weilin Zhong, Hongda Yin, Zhengdong Luo, Biao Luo, Bin Liang, Min He, Junjie Huang

**Affiliations:** 1 Yueyang City Roads and Bridge Construction Corporation, Yueyang 414021, China; huyong02@sohu.com (Y.H.); zhongwl123@163.com (W.Z.); yinhd0101@sohu.com (H.Y.); 2 Department of Transportation of Hunan Province, Traffic Manufacturing Cost Management Station, Changsha 410116, China; chenshanling001@sohu.com; 3 College of Civil Engineering and Mechanics, Xiangtan University, Xiangtan 411105, China; cekunxie11@sohu.com; 4 College of Civil Engineering, Hunan University of Technology, Zhuzhou 412007, China; liangbingg123@sina.com (B.L.); heemingg@163.com (M.H.); 5 China Construction Technology of Hunan Co., Ltd., Changsha 410600, China; huangjunjiee@aliyun.com

**Keywords:** bamboo grid, soil–rock mixture, subgrade, pull-out test, bamboo reinforcement

## Abstract

To promote the application of the bamboo grid in the soil–rock mixture subgrade in mountain areas, the mechanical properties of bamboo reinforcement were investigated in this study, and the reinforcement effect and interface characteristics of uniaxial/biaxial bamboo grid on the soil–rock mixture under different vertical loads was comparatively analyzed. The results show that the tensile force (2% elongation) of the bamboo reinforcement is 50.21 kN/m, and its average tensile strength is 236.01 MPa. Moreover, bamboo reinforcement has excellent shear and flexural properties. In general, the reinforcement effect of the biaxial bamboo grid on the soil–rock mixed subgrade is better than that of the uniaxial bamboo grid. In the case of using a uniaxial bamboo grid, its pull-out curve is generally a strain-softening type. As for the biaxial bamboo grid, due to the existence of bite force, its pull-out curve usually presents a strain-hardening type. Compared with the uniaxial bamboo grid, the friction coefficient of the reinforcement–soil interface using the biaxial bamboo grid is higher, and the interfacial shear stress is increased by 72.2–91.2%.

## 1. Introduction

Highways are inevitably distributed in mountain areas and foothills [1,2], and their construction is restricted by complex geography. The soil–rock mixture produced by mountain excavation, as subgrade filler, has gradually become the main construction form of mountain highway construction [3,4,5]. The soil–rock mixture is a kind of special filler composed of stones and soil, and its properties are different from cataclastic rock and homogeneous soil [6,7,8]. Existing studies have shown that due to the characteristics of large particle size, the high content of coarse particles, and inhomogeneity in the soil–rock mixture, it is difficult to compact or unevenly compact in subgrade construction [9,10,11,12,13]. Defects such as lateral spreading, breakage, and differential settlement occur due to the inhomogeneity and granularity of the soil–rock mixture, under the upper load. These problems will reduce the safety of highway operations and increase maintenance costs, thereby inhibiting regional economic development. Horizontal reinforcement is one of the effective methods that can be used to solve the above problems.

Geosynthetics as reinforcing elements are a cost-effective solution for improving the load-bearing properties of granular fill subgrade. The most representative of them is the geogrid, which is often used as the horizontal reinforcement material of the subgrade. It can significantly improve the overall stability and horizontal deformation control of the subgrade structure [14,15,16]. According to the different types of weaving, geogrids are divided into uniaxial, biaxial, and multiaxial [17,18]. Liu et al. [19] laid a geogrid inside the fill to form a load-bearing composite, which effectively restrained the lateral deformation and improved the overall stress state of the soil. Sweta et al. [11] found that the utilization of geogrids directly enhanced the shear strength of ballast and reduced the percentage of breakage by 9.24%. Esmaeili et al. [20] reported that the number of geogrid layers is one of the important factors affecting the lateral resistance of a single sleeper/track panel. In another study, Gao et al. [21] performed model tests based on transparent soil technology and digital image technology to comprehensively study the effect of the layers and types of geogrids on the reinforcement effect of foundation soil. In addition, due to the excellent tensile properties of the geogrid, it can also be used as the wrapping material of the gravel pile to limit the lateral deformation of the gravel pile. In addition, due to the excellent tensile properties of the geogrid, it can be used as the wrapping material of the gravel pile to limit its lateral deformation [22,23,24]. However, the widely used geosynthetic material is a chemical product made of a high molecular polymer as a raw material, which is a non-renewable resource. Additionally, its production process will produce a large amount of sulfide, nitrogen oxides and carbon dioxide, and other gases, which will pollute the environment [25]. At present, the construction industry advocates green and low-carbon development and searches for sustainable reinforcement materials similar to geogrids, which have important theoretical and environmental significance in the field of soil–rock mixture reinforcement.

Bamboo is a natural biomaterial with high strength, high toughness, and renewable, and is easily accessible in Asia. It is also widely distributed in mountain areas where highway construction is carried out. In China, the growing area of bamboo has reached 7.2 million hectares. Bamboo can be used in civil engineering simply by optimizing its anti-corrosion ability of bamboo. Generally, bamboos are mostly used for auxiliary structures and members, but some are also used as a part of load-bearing structures. Several studies have confirmed the ability of bamboo as a reinforcement material in composite panels, concrete elements, and shear connectors, and put forward the idea of large-scale application [26,27,28,29,30]. In addition, the biggest advantage of bamboo is its high tensile properties. It is estimated that the tensile strength and elastic modulus of bamboo grids are about 20 times and 30 times that of geogrids, respectively, while the elongation is only 1/6 of those of geogrids. Therefore, the bamboo grid woven with bamboo reinforcements instead of geogrids has broad application prospects in geotechnical engineering [31,32,33]. Hegde et al. [34] performed model tests and found that the combination of geocells and geogrids increased the bearing capacity of the clay bed by six times, and the settlement and deformation of the foundation were effectively controlled. Ahirwar et al. [35] found that parameters such as laying position, grid size, number of reinforcement layers, and grid shape all affect the bearing capacity of the foundation reinforced with the bamboo grid.

All the above studies report the applicability of bamboo as an alternative material for geogrid/rebar. Moreover, the research on the reinforcement of the foundation by bamboo grids is basically carried out on the soft clay/sand layer system with few coarse particles and well graded. However, there are few experimental studies on the improvement of special soil-rock mixture subgrades with the bamboo grid. In order to promote the application of bamboo grids in the reinforcement of soil-rock mixture, this study mainly investigated the tensile, shear, and flexural properties of bamboo reinforcement. Further, a field pull-out test was performed to study the interfacial friction properties of uniaxial/biaxial bamboo grid reinforced soil–rock mixtures under different vertical loads.

## 2. Mechanical Properties Test of Bamboo Reinforcement

The premise of using bamboo reinforcement as a subgrade reinforcement material is to ensure that its strength meets the performance requirements required by actual working conditions. Previous studies have shown that the mechanical properties of bamboo are closely related to the growth years of Moso bamboo, and the bamboo strength gradually increases with time in the early growth period (within 3 years). When the bamboo age reaches 3 to 5 years, the bamboo strength reaches its maximum value and tends to be stable. After that, bamboo gradually loses its strength with time. From this, a 3-year Moso bamboo with a diameter of 40–60 mm and a thickness of 8–12 mm was selected for this study.

### 2.1. Parallel-to-Grain Tensile Strength

**Figure 1:**
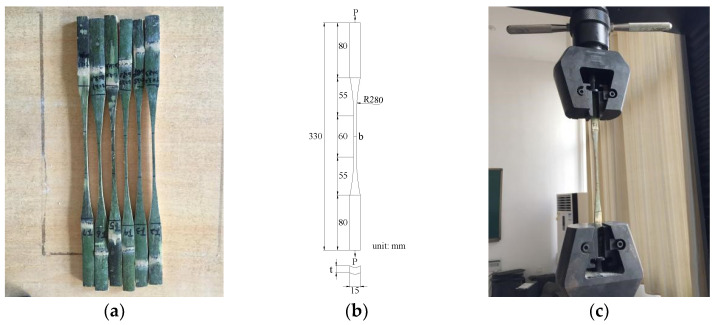
Tensile test specimens and instruments: (**a**) tensile test specimens; (**b**) dimensional information; (**c**) tensile test.

The tensile strength is mainly controlled by bamboo age, size, grain, etc. In order to reduce interference factors, we prepared bamboo reinforcements along the grain that was consistent with the bamboo growth direction. The fabrication of tensile test specimens and the design of test procedures were carried out in accordance with JG/T 199-2007 [36]. According to the specification [36], six specimens were prepared for the test, as shown in Figure 1a. The size of the tensile test specimen is detailed in Figure 1b, where “*b*” is the effective width and “*t*” is the thickness. The prepared specimens were performed by a CMT5105 universal testing machine (Figure 1c), and the loading rate was 1 mm/min.

The specific tensile strength results are shown in Table 1. The tensile force of the bamboo reinforcement is 50.21 kN/m, corresponding to 2% elongation, and its average tensile strength is 236.01 MPa.

### 2.2. Shear Strength

**Figure 2:**
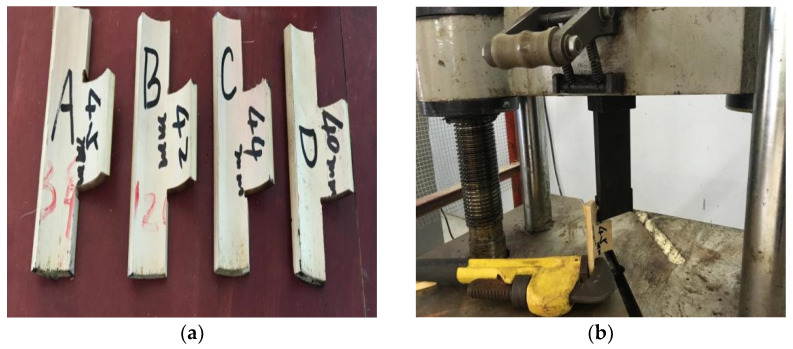
Shear test specimens and instruments: (**a**) shear test specimens; (**b**) shear test.

In order to gain a detailed understanding of the mechanical properties of bamboo reinforcements and determine appropriate reinforcement sizes, this study also investigated the shear strength of bamboo reinforcements. As shown in Figure 2a, a total of four specimens with the same thickness (10 mm) and different shear lengths (40, 42, 44, and 45 mm) were prepared. The shear strength of the bamboo reinforcement was tested using a WE-100B universal testing machine with a maximum load of 100 kN. The bottom of the specimen was fixed by steel bar clamps as a fixture to ensure that the bulging part of the specimen was used as a bearing part (Figure 2b).

The shear strength of bamboo reinforcement increases roughly linearly with increased shear length, based on the test results (shown in Table 2). This is because the increase in shear length is equivalent to increasing the content of vascular bundles in bamboo reinforcement, which evidently facilitates growth in strength.

### 2.3. Flexural Strength

**Figure 3:**
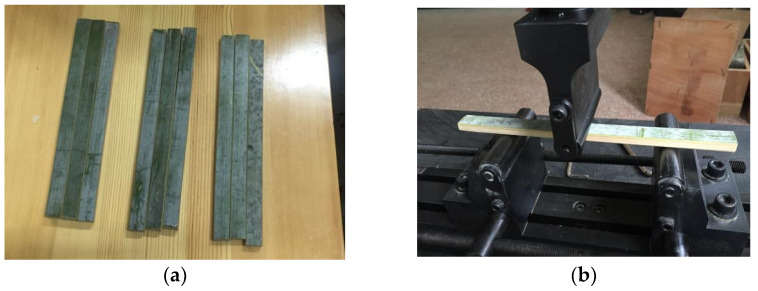
Flexural test specimens and instruments: (**a**) flexural test specimens; (**b**) flexural test.

According to the specification [36], the Moso bamboo was processed along the grain into three types of bamboo reinforcements with a length of 220 mm and a width of 20 mm, and their thicknesses were 8, 10, and 12 mm, respectively. Three specimens were made for each type of bamboo reinforcements, totaling nine specimens, as shown in Figure 3a. The test instrument used was a CMT5105 universal testing machine, which was consistent with the tensile strength test. As shown in Figure 3b, the three-point loading method was used in this test, and the loading rate was set to 1 mm/min.

In this study, Formula (1) is used to calculate the flexural strength of bamboo reinforcement, and the average value of the strength of the three specimens is taken as the representative value.

(1) $$f_{f}=\frac{3PL}{2bt^{2}}$$

In Formula (1), *f_f_* is the flexural strength of the bamboo reinforcement (unit MPa); *P* is the failure load (unit N); *L* is the span (unit mm); *b* is the width of the bamboo reinforcement (unit mm); *t* is the thickness of the bamboo reinforcement (unit mm).

As shown in Table 3, the flexural strengths of bamboo reinforcements with thicknesses of 8, 10, and 12 mm were 117.49, 123.87, and 121.26 MPa, respectively. This shows that the bamboo reinforcement with a thickness of 10 mm obtained the maximum flexural strength value. Based on this, bamboo reinforcements with a thickness of 10 mm and a width of 20 mm will be used as the weaving material.

### 2.4. Comparison of Mechanical Properties between Bamboo Reinforcement and Uniaxial Geogrid

The obtained bamboo reinforcement strength is compared with the uniaxial geogrid (TGDG80 and TGDG120) strength in GB/T 17689-2008 [37], as shown in Table 4. As observed, the tensile force index value (2% elongation) of bamboo reinforcement meets the requirements of the specification [37]. What is more, bamboo reinforcement has excellent tensile, shear, and flexural properties, which are not available in those uniaxial geogrids. This confirms that bamboo reinforcement has great application potential as a reinforcement material for subgrade.

## 3. Field Pull-Out Test

Compared with traditional geogrids, bamboo materials with high strength and easy availability have broad application prospects in the reinforcement of soil–rock mixture subgrade. Previous studies mostly used indoor pull-out tests, and the test results were limited by interference factors: (1) a part of the vertical load was consumed by the frictional forces on the side wall of the test chamber. As a result, the pull-out force of the bamboo grid was underestimated. (2) The size design of the bamboo lattice results in a scaling effect. To reduce the test error, a field pull-out test was carried out. The purpose of this study is to investigate the friction characteristics of the reinforced soil interface of the uniaxial/biaxial bamboo grid reinforced soil–rock mixture subgrade under different vertical loads. The uniaxial bamboo grid refers to bamboo reinforcement.

### 3.1. Soil–Rock Mixture Material

The subgrade filler used in this study was the soil–rock mixture produced in actual construction. It was composed of clay and stone crushed by the mountain. It was determined that the proportion of stones in the soil–rock mixture was 58%, the uniformity coefficient (C_u_) was 15, and the curvature coefficient (C_c_) was 1.69, based on the specific gradation (shown in Table 5). The soil–rock mixture was filled in layers and compacted by a road roller to ensure that the compaction degree of each layer of soil–rock mixture subgrade reached 96–98%.

### 3.2. Antiseptic Treatment of Bamboo Reinforcement and Preparation of Bamboo Grid

Bamboo, a natural biocomposite, faces durability challenges as a permanent building material. Affected by biodegradation, natural decay occurs when bamboo is buried in the soil for a long time. Dang et al. [38] found that, based on the consolidation theory, the soil has reached deformation stability before the bamboo reinforcement fails (the natural corrosion age of untreated bamboo reinforcement is about 3–5 years). In order to prolong the service life of bamboo reinforcement and enhance the long-term bearing performance of subgrade, many scholars have used chemical solution soaking to pretreat bamboo. Existing studies have confirmed that the treated bamboo reinforcements can effectively resist fungal attacks and insects [27,34,39,40]. In this study, bamboo reinforcements were soaked in a 5% copper–chrome–arsenic solution for 7 days. After that, the bamboo reinforcements were placed vertically in a dry environment to air dry for 7 days.

The bamboo reinforcements used were segmented manually along the grain of the Moso bamboo. The dimensions of the bamboo reinforcements were 2.5 m in length, 20 mm in width, and 10 mm in thickness. All biaxial bamboo grids were weaved manually into an orthogonal arrangement to keep the symmetry for pull-out testing. The aperture size of their inner square aperture is always 10 cm × 10 cm.

### 3.3. Test Procedure

The test is divided into two different working conditions: the uniaxial bamboo grid (W1 condition) and the biaxial bamboo grid (W2 condition) made of orthogonally arranged bamboo reinforcement. The interfacial shear stress and friction coefficient of reinforced soil under vertical loads of 20, 40, 60, 80, and 100 kPa were mainly measured, so as to compare and analyze the reinforcement effect and mechanism of uniaxial/biaxial bamboo grids in soil–rock mixture subgrade. The vertical load took the form of graded stacking, and its size was determined by the thickness of the upper soil–rock mixture filler. Hydraulic jacks were used to apply horizontal loads, controlled by the slow sustaining load method.

### 3.4. Results and Discussion

In the pull-out test of the geogrid, Formula (2) is used to calculate the interface shear stress (*τ_P_*) of the grid. Since the thickness of the bamboo grid in this study is several times that of the geogrid, the friction between its side and the soil cannot be ignored. Based on the actual working conditions, Formula (3) is obtained by optimizing Formula (2). The interfacial friction coefficient (*f*) of the reinforced soil is calculated according to Formula (4).

(2) $$\tau_{P}=\frac{T_{P}}{2bl^{\prime }}$$

(3) $$\tau_{P}=\frac{T_{P}}{2bl^{\prime }+2tl^{\prime }}$$

(4) $$f=\frac{\tau_{P}}{p}$$

In Formulas (2) and (3), *T_P_* is the pull-out force (unit kN); *b*, *l*′, and *t* are the width, effective length, and thickness of the bamboo reinforcement, respectively, unit m. Regardless of the working conditions, the pull-out force acts directly on a single bamboo reinforcement. Therefore, the calculation variables of working condition 2 take the same values as those of working condition 1. Specifically, *b*, *l*′, and *t* are 0.02, 2.5, and 0.01 m, respectively. In Formula (4), *p* is the vertical load (unit kPa).

#### 3.4.1. Working Condition of Soil–Rock Mixture-Uniaxial Bamboo Grid

**Figure 4:**
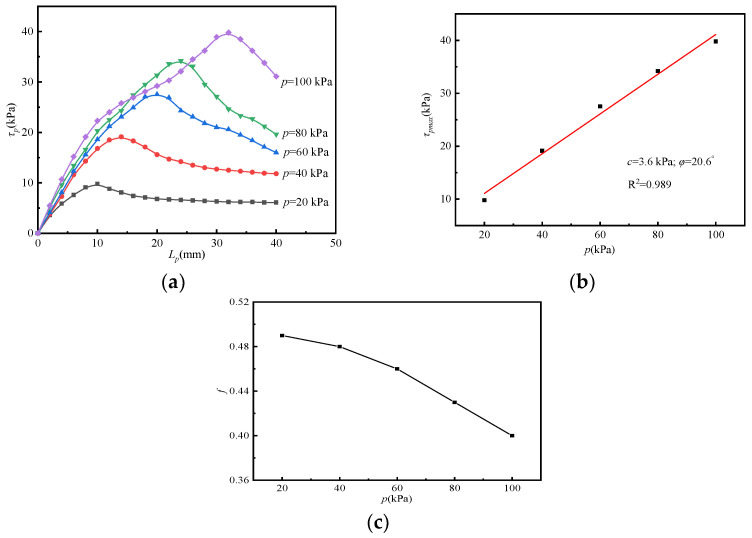
Relationship curves under W1 condition: (**a**) *τ_p_*-*L_p_*; (**b**) *τ_pmax_*-*p*; (**c**) *f*-*p.*3.4.2. Working condition of soil–rock mixture biaxial bamboo grid.

The effect of vertical load (*p*) on interface properties is considered in the pull-out test of the soil–rock mixture uniaxial bamboo grid. Figure 4a–c is the interfacial shear stress-pulling displacement (*τ_p_*-*L_p_*), maximum shear stress vertical load (*τ_pmax_*-*p*), and friction coefficient vertical load (*f*-*p*) relationship curves obtained under different vertical load conditions.

From Figure 4a, it is found that under different vertical loads, the *τ_p_*-*L_p_* curve enters a descending stage after the shear stresses reach their peak value. Apparently, the pull-out curve exhibits a significant strain-softening feature. As observed, the maximum shear stresses are 9.80, 19.13, 27.53, 34.20, and 39.80 kPa when the vertical loads are 20, 40, 60, 80, and 100 kPa, respectively. Obviously, the magnitude of the vertical load not only affects the peak value of shear stress but also affects the displacement when the peak value occurs. During the pull-out test, the shear stress of the reinforced soil interface gradually changes synergistically with increased pull-out displacement, based on the mechanism analysis. Ultimately, the increased vertical load applied enhances the interfacial confinement of the reinforced soil. A linear fit was performed to the *τ_pmax_*-*p* relationship shown in Figure 4b. It can be obtained that the interfacial cohesion (*c*) and the friction angle (*φ*) are 3.6 kPa and 20.6°, respectively, and the correlation coefficient (R^2^) of the linear fit is 0.989.

The interfacial friction coefficient can reflect the friction and shear characteristics between different materials. As observed in Figure 4c, the friction coefficient between the soil–rock mixture uniaxial bamboo grid decreases roughly linearly with the increased vertical load. The friction coefficient of the reinforced soil interface under the vertical load of 40 kPa decreases by 2.38% compared with that of 20 kPa. Further, when the vertical load is increased to 60 kPa, the friction coefficient is reduced by 4.07% compared with that of 40 kPa. It shows that this decreasing trend becomes more significant with the increase in vertical load.

#### 3.4.2. Working Condition of Soil–Rock Mixture-Biaxial Bamboo Grid

**Figure 5:**
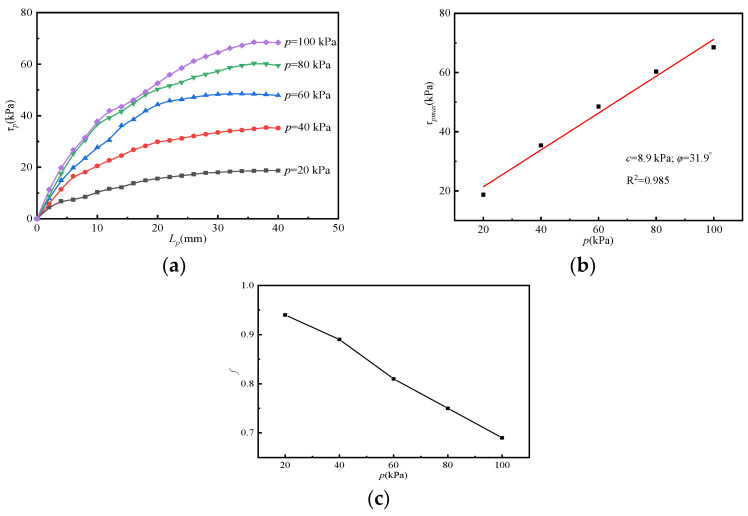
Relationship curves under W2 condition: (**a**) *τ_p_*-*L_p_*; (**b**) *τ_pmax_*-*p*; (**c**) *f*-*p.*

To compare and analyze the reinforcement effect and different action mechanisms of the uniaxial/biaxial bamboo grid in soil–rock mixture filler, a group of pull-out tests involving soil–rock mixture biaxial bamboo grid was carried out. The specific test results are shown in Figure 5a–c.

It can be seen from Figure 5a that the shear stress of the reinforced soil interface is significantly affected by the vertical load and is positively correlated. When the vertical loads are 20, 40, 60, 80, and 100 kPa, the maximum shear stresses are 18.73, 35.40, 48.53, 60.33, and 68.53 kPa, respectively. In contrast, the maximum shear stress of the biaxial bamboo grid condition (W2) increases by 91.2%, 85.0%, 76.3%, 76.4%, and 72.2%, respectively, compared with the uniaxial bamboo grid condition (W1). The variation of the maximum shear stress under different vertical loads is plotted as shown in Figure 5b, and a linear fit is performed. It can be obtained that the interfacial cohesion (*c*) and the friction angle (*φ*) are 8.9 kPa and 31.9°, respectively, and the correlation coefficient (R^2^) of the linear fit is 0.985. Comparing the test results in Section 3.4.2, it can be found that the utilization of the biaxial bamboo grid significantly increases the interfacial cohesion (increased by 147.2%) and the friction angle. The interfacial friction coefficient of W2 is also significantly higher than that of W1.

In general, the reinforcement effect of the biaxial bamboo grid on the soil–rock mixed subgrade is better than that of the uniaxial bamboo grid. The interfacial shear stress is mainly controlled by the surface friction between the grid and the filler. However, the bite force created by partial filler and the grille square apertures also plays an important role. Comparing Figure 4a and Figure 5a, it can be seen that, different from the strain-softening phenomenon exhibited by W1, the pull-out curve of W2 exhibits a slight strain-hardening feature. This is because the uniaxial bamboo grid is mainly affected by surface friction. For the biaxial bamboo grid, the existence of transverse ribs enhances the impedance, and the resulting interlocking occlusion gradually increases with the increased displacement. On the macroscopic level, it is manifested as higher interfacial cohesion, and the interfacial friction coefficient increases accordingly.

## 4. Summary and Conclusions

The soil–rock mixture is a kind of special filler composed of stones and soil. Defects such as lateral spreading, breakage, and differential settlement occur due to the inhomogeneity and granularity of the soil–rock mixture under the upper load, thus affecting the safety of highway operations and the development of a regional economy. Natural biological material such as bamboo grid for horizontal reinforcement is one of the effective methods used to solve the above problems. In view of this, this study tested the tensile, shear, and flexural properties of bamboo reinforcements to evaluate the feasibility of bamboo as reinforcement materials. In the field pull-out test, the reinforcement effect of the uniaxial/biaxial bamboo grid in the soil–rock mixture filler and the exertion mechanism of the interfacial shear stress were comparatively analyzed, and the influence of vertical load (20, 40, 60, 80, and 100 kPa) changes on interfacial parameters was discussed. The following valuable conclusions can be drawn:

(1) The tensile force (2% elongation) of the bamboo reinforcement is 50.21 kN/m, and its average tensile strength is 236.01 MPa. At the same time, bamboo reinforcement has excellent shear and flexural properties. This confirms that bamboo reinforcement has great application potential as a reinforcement material for subgrade.
(2) Different from the geogrid, the shear stress between the bamboo reinforced grid and the soil is affected by the surface friction and the side friction. The friction force between the uniaxial bamboo grid and the soil–rock mixture filler plays a dominant role in the interfacial shear strength, and the pull-out curve generally shows a strain-softening type. For the biaxial bamboo grid, due to the existence of the internal square apertures, the bite force between the filler and the reinforcement significantly affects the interfacial shear strength. The bite force gradually develops as the displacement increases, and the pull-out curve usually exhibits a strain-hardening type. The magnitude of the vertical load not only affects the peak value of shear stress but also affects the displacement when the peak value occurs.
(3) Compared with the uniaxial bamboo grid, the use of the biaxial bamboo grid shows a higher friction coefficient of the reinforcement soil interface. In contrast, the reinforcement effect of the biaxial bamboo grid is more significant. When the biaxial bamboo grid is used, the interfacial cohesion is increased from 3.6 kPa (uniaxial) to 8.9 kPa (biaxial), and the friction angle is increased from 20.6° (uniaxial) to 31.9° (biaxial).

## Figures and Tables

**Table 1:** Tensile strength test results.

Label	*b*/mm	*t*/mm	Calculation Formula	*P*/N	*f_t_*/MPa
T1	4.12	10.28	$f_{t}=\frac{P}{bt}$ where “*f_t_*” is the tensile strength (unit MPa) and “*P*” is the failure load (unit N).	9280	219.11
T2	3.95	9.62		9760	256.85
T3	3.86	9.77		9630	255.36
T4	4.05	10.22		8810	212.85
T5	4.15	10.16		1046	232.09
T6	3.88	10.18		8840	223.81
Average value	4.00	10.04	-	9463	236.01

**Table 2:** Shear strength test results.

Label	*l*/mm	*t*/mm	Calculation Formula	*P*/N	*f_s_*/MPa
S1	40	10	$f_{s}=\frac{P}{lt}$ where “*f_s_*” is the shear strength (unit MPa) and “*l*” is the shear length (unit mm).	4236	10.59
S2	42	10		4510	10.74
S3	44	10		4870	11.07
S4	45	10		5127	11.40

**Table 3:** Flexural strength test results.

*t*/mm	8	10	12
***f_f_*** **/MPa**	117.49	123.87	121.26

**Table 4:** Strength comparison results.

Type	Tensile Force (at 2% Elongation)/kN·m^−1^	*f_t_*/MPa	*f_s_*/MPa	*f_f_*/MPa
Bamboo reinforcement	50.21	236.01	10.59	123.87
TGDG80	≥26.0	-	-	-
TGDG120	≥36.0	-	-	-

**Table 5:** Gradation of soil–rock mixture.

Fraction/mm	0–2	2–5	5–10	10–20	20–40	40–60
**Content/%**	24	18	27	16	8	7

## Data Availability

Not applicable.
